# Supplementary material for: Novel Driver Strength Index highlights important cancer genes in TCGA PanCanAtlas patients
Source: PeerJ. 2022 Aug 11;10:e13860. doi: 10.7717/peerj.13860 (PMC9375969; doi:10.7717/peerj.13860)
Supplement: Supplemental Information 2 [file peerj-10-13860-s002.zip › COHORTS/genes plots/detailed/2021_8_16_14_9_distribution_events_detailed_17.pdf]

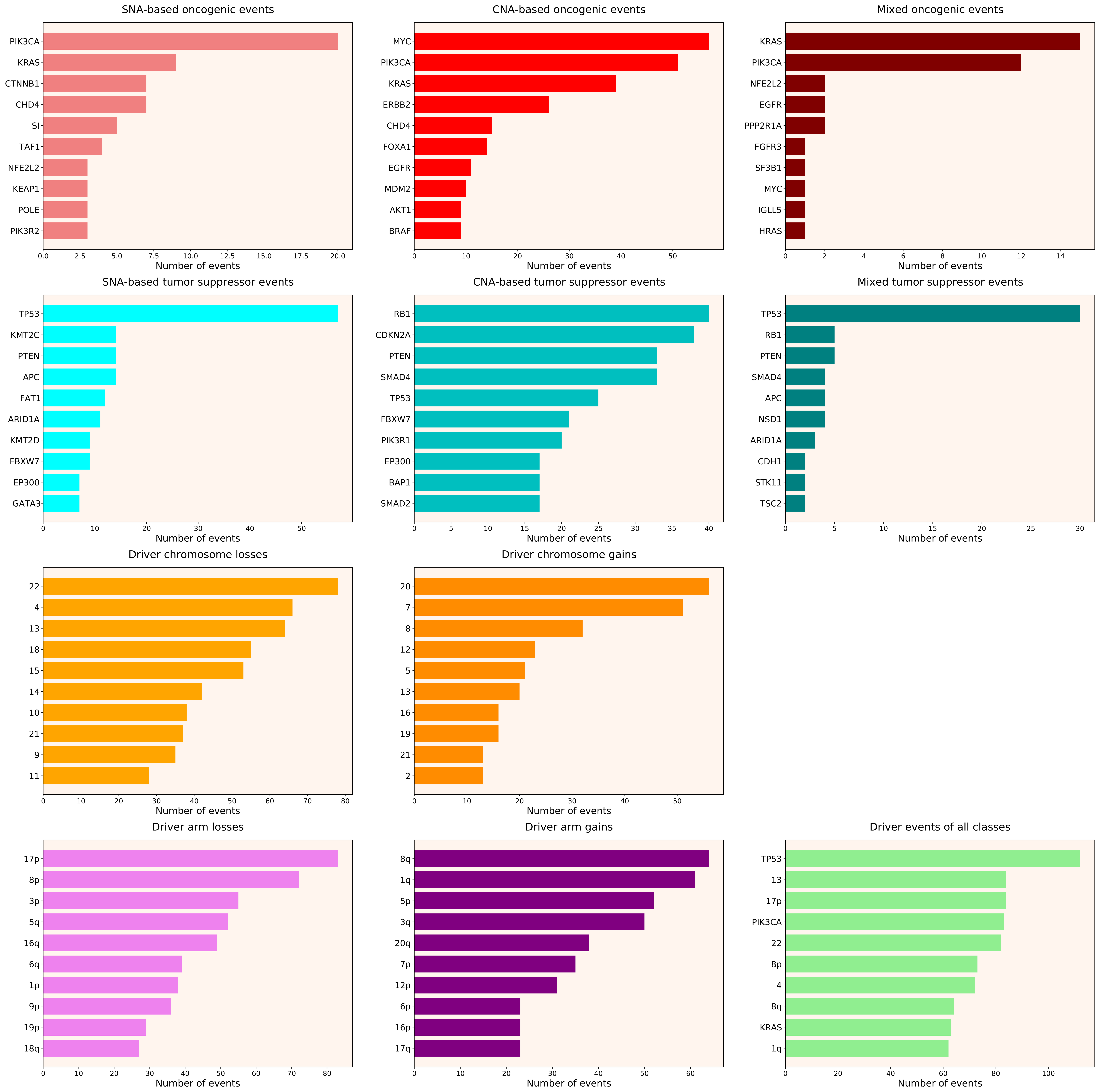
SNA-based oncogenic events
PIK3CA
KRAS
CTNNB1
CHD4
SI
TAF1
NFE2L2
KEAP1
POLE
PIK3R2
0.0 2.5 5.0 7.5 10.0 12.5 15.0 17.5 20.0
Number of events
CNA-based oncogenic events
MYC
PIK3CA
KRAS
ERBB2
CHD4
FOXA1
EGFR
MDM2
AKT1
BRAF
0 10 20 30 40 50
Number of events
Mixed oncogenic events
KRAS
PIK3CA
NFE2L2
EGFR
PPP2R1A
FGFR3
SF3B1
MYC
IGLL5
HRAS
0 2 4 6 8 10 12 14
Number of events
SNA-based tumor suppressor events
TP53
KMT2C
PTEN
APC
FAT1
ARID1A
KMT2D
FBXW7
EP300
GATA3
0 10 20 30 40 50
Number of events
CNA-based tumor suppressor events
RB1
CDKN2A
PTEN
SMAD4
TP53
FBXW7
PIK3R1
EP300
BAP1
SMAD2
0 5 10 15 20 25 30 35 40
Number of events
Mixed tumor suppressor events
TP53
RB1
PTEN
SMAD4
APC
NSD1
ARID1A
CDH1
STK11
TSC2
0 5 10 15 20 25 30
Number of events
Driver chromosome losses
22
4
13
18
15
14
10
21
9
11
0 10 20 30 40 50 60 70 80
Number of events
Driver chromosome gains
20
7
8
12
5
13
16
19
21
2
0 10 20 30 40 50
Number of events
Driver arm losses
17p
8p
3p
5q
16q
6q
1p
9p
19p
18q
0 10 20 30 40 50 60 70 80
Number of events
Driver arm gains
8q
1q
5p
3q
20q
7p
12p
6p
16p
17q
0 10 20 30 40 50 60
Number of events
Driver events of all classes
TP53
13
17p
PIK3CA
22
8p
4
8q
KRAS
1q
0 20 40 60 80 100
Number of events
